# Clinical plasma cells-related genes to aid therapy in colon cancer

**DOI:** 10.1186/s12864-023-09481-4

**Published:** 2023-08-01

**Authors:** Qi Zhang, Xiao Feng, Mingming Zhang, Wenjing Sun, Yuqing Zhai, Shuangshuang Qing, Ying Liu, Haoran Zhao, Jing Sun, Yi Zhang, Chaoqun Ma

**Affiliations:** 1 Department of General Surgery, Jiangsu Province Hospital of Chinese Medicine, Affiliated Hospital of Nanjing University of Chinese Medicine, Nanjing, 210029 China; 2 Zhuzhou Orthopaedic Hospital of Traditional Chinese Medicine, Zhuzhou, 412000, China; 3 Xi’an Daxing Hospital, Xian, 710000 China; 4 Department of Oncology, The First Affiliated Hospital of Nanjing Medical University, Nanjing, 210029 China

**Keywords:** Colon cancer, Plasma cells, Tumor immune microenvironment, Prognosis prediction, Clinical therapy, Tumor mutation burden

## Abstract

The tumor immune microenvironment (TIME) of colon cancer (CC) has been associated with extensive immune cell infiltration (IMI). Increasing evidence demonstrated that plasma cells (PC) have an extremely important role in advance of antitumor immunity. Nonetheless, there is a lack of comprehensive analyses of PC infiltration in clinical prognosis and immunotherapy in CC. This study systematically addressed the gene expression model and clinical information of CC patients. Clinical samples were obtained from the TCGA (The Cancer Genome Atlas) databases. Gene ontology (GO), Kyoto Encyclopedia of Genes and Genomes (KEGG), GSVA, and the MAlignant Tumors using Expression data (ESTIMATE) algorithm were employed to research the potential mechanism and pathways. Immunophenoscore (IPS) was obtained to evaluate the immunotherapeutic significance of risk score. Half maximal inhibitory concentration (IC50) of chemotherapeutic medicine was predicted by employing the pRRophetic algorithm. A total of 513 CC samples (including 472 tumor samples and 41 normal samples) were collected from the TCGA-GDC database. Significant black modules and 313 candidate genes were considered PC-related genes by accessing WGCNA. Five pivotal genes were established through multiple analyses, which revealed excellent prognostic. The underlying correlation between risk score with tumor mutation burden (TMB) was further explored. In addition, the risk score was obviously correlated with various tumor immune microenvironment (TIME). Also, risk CC samples showed various signaling pathways activity and different pivotal sensitivities to administering chemotherapy. Finally, the biological roles of the *CD177* gene were uncovered in CC.

**Supplementary Information:**

The online version contains supplementary material available at 10.1186/s12864-023-09481-4.

## Introduction

Colon cancer (CC) is the third most frequent malignancy and the second most common cause of cancer-related death worldwide [1]. Radical surgery remains the major scheme therapy for non-metastatic CC. However, recurrence after surgery remains a problem to resolve. It has been reported that 80% of recurrence occurs in the first 3 years after therapy, most commonly resulting in death [2]. Also, the median survival time for patients with distant metastases is only 10–12 months, even after palliative care and treatment [3]. Hence, for advanced patients who missed the opportunity for curable surgery, systematic or multidisciplinary treatment strategies, such as chemotherapy, targeted treatment, and immunotherapy, may be considered to enhance the prognosis [4, 5].

Accumulating evidence indicated a large number of PC infiltration in cancer [6], and several studies provided evidence that the higher expression of PC metagene is associated with excellent prognosis in some cancers [7, 8]. In their study, Chen-Kian et al*.* demonstrated that inhibitors secreted by PC are necessary to terminate the tumor cell cycle [9].

Overall survival (OS) was found to be improved in several tumors following the use of immune checkpoint blockade (ICB) therapy [10–12]. While the role of PC in local tumor immunity is unclear, tumor-specific IgG1 antibodies produced through PC are known to exert antitumor effects via antibody-dependent cytotoxicity [13]. Another study demonstrated that PC in breast cancer overexpress PD-L1, and there is also evidence of direct interaction between PC and immune cells [14]. Besides, PD-L1 PC has been reported to inhibit helper T and B lymphocytes by generating IL-10 [15]. Accumulating evidence shows that PC occupies a significant position in tumor immunity.

Currently, there is no all-encompassing analysis of the biological role of PC in colon cancer prognosis and tumor microenvironment. Therefore, immune profiling may be the most reliable and promising strategy for omnidirectional evaluation of tumor susceptibility to clinical treatment, used to identify CC cases based on specific risk profiles associated with PC profiling and generate individualized procedures to improve efficacy accordingly. Accordingly, in the present study, we employed the TCGA CC sample dataset to explore the underlying role of PC profiling. PC features were then obtained by the CIBERSORT algorithm, and the most available black module associated with PC was detected using WGCNA [16, 17]. Five hub genes and multiple-COX regression models were recognized. Consequently, a multi-genes risk model and a comprehensive prognostic nomogram were constituted. Finally, the synergistic effect of risk score with TMB was identified. In addition, the underlying role of the risk score in TIME [18] was explored. The potential therapeutic prediction and signaling pathways of risk score were uncovered.

**Fig. 1:**
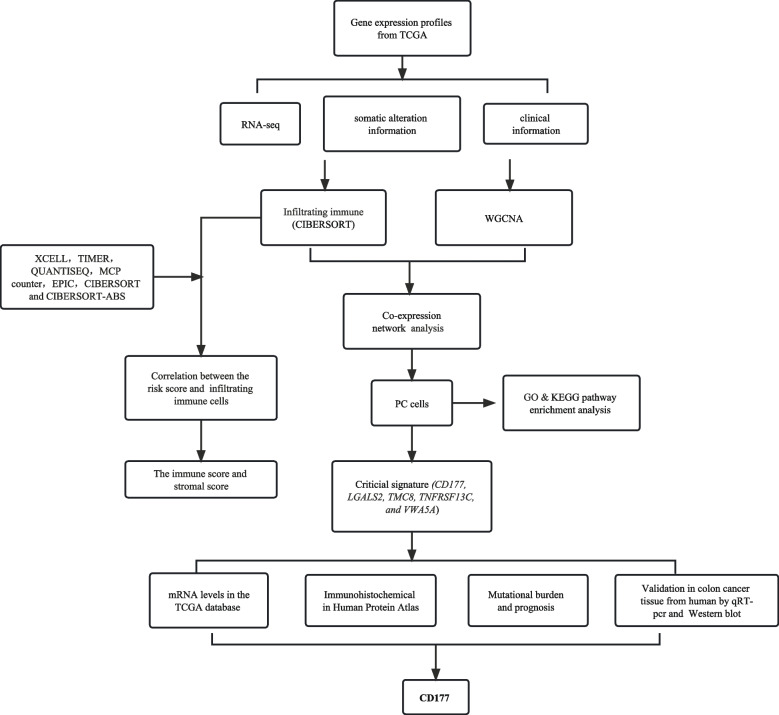
Flowchart of the study

*CD177* is considered to have an important role in affecting the clinical and prognostic value of various cancers. For example, overexpression of *CD177* has been associated with detrimental outcomes in ovarian and pancreatic ductal adenocarcinoma and excellent outcomes in breast cancer [19, 20]. More importantly, current research has demonstrated that *CD177* could improve the inhibitory function of tumor-infiltrating Treg cells (TC) in the TME. In addition, blocking *CD177* with antibodies in *CD177* + tumor-infiltrating cells may be a novel target for antitumor immunotherapy [21]. *CD177* has been addressed by many tumor-related studies [20, 22–24], and some experiments have verified its expression differences. The above shows that *CD177* may be closely related to the occurrence and development of tumors. More importantly, we found that *CD177* had the most stable and significant differential expression in human CC tissue samples among the five key genes screened. However, the biological function of *CD177* in CC remains unclear, so we focused on selecting CD177 for further verification and research. In addition, the biological functions of *CD177* in predicting prognosis, immunotherapy, and immune infiltration of CC were investigated to provide a strong perception of clinical CC treatment strategies Fig. 1.

## Materials and methods

### Data collection

CC-associated mRNA sequences and clinical data were used from TCGA-GDC (https://portal.gdc.cancer.gov/), type, including RNA-seq, clinical information and somatic alteration information. We obtained transcriptome profiles of 472 tumor samples and 41 normal samples. Incomplete data were excluded. All data were pre-processed by Perl language and R software.

### The landscape of infiltrating immune cells

The CIBERSORT algorithm (http://cibersort.stanford.edu/) was used to analyze and calculate the sequencing data of the samples and the abundance of 22 tumor-infiltrating immune cells (TIC), which were obtained based on the cellular composition of the TIME [25].

### Weighted gene co-expression network analysis

Sequencing data of 11,283 genes from CC patients were used to produce weighted co-expression networks by the WGCNA [17] to identify core modules associated with characteristic immune cell subtypes in CC patients. Next, the power value scatter plot was created, and optimal soft power value (soft power = 0.9) was selected to obtain the correlation matrix between genes, cluster the genes, dynamically identify and cut the module (minModules Size = 60), plot the pattern of the gene module, and find and merge similar modules to obtain the module graph. A heat map was used to assess the correlation between the module and immune cells based on the immune cell results file. The samples were filtered according to *p* < 0.05; the correlation coefficient was obtained by correlation testing, and the correlation heat map was drawn. Next, each module was cycled to obtain the genes contained in each module. Finally, the "PC" population was emphasized, and the modules most correlated to PC were extracted for subsequent analysis.

### Functional enrichment analysis

The Entrez ID of each PC-associated gene was acquired through the R package " org.Hs.eg.db [26] ". KEGG [27–30] and GO [31] pathway annotation were performed through "clusterProfiler [32] ", "enrichplot [33] " and "ggplot2 [34] " packages to demonstrate the potential mechanism of hub gene in biological processes associated with PC and visualize the results [35]. The samples were grouped according to the expression amount of the target gene, and the logFC of the high and low expression groups was obtained. Then, the genes were sequenced and analyzed. According to the *p*-value and the corrected *p*-value as the filter conditions, the results of significant enrichment were obtained and visualized (termNum = 8). In addition, the expression data were analyzed and scored by GSVA. Finally, the normal sample was removed, the tumor sample and the risk set were intersected to obtain the intersection sample, the gene set was circulated to obtain the expression of the gene and gene set, and the correlation test was performed (*p*-value < 0.001***, *p*-value < 0.01**, *p*-value < 0.05*).

### Construction and validation of PC-related prognostic signature

The most important module of genes was implemented to construct prognostic risk profiles for CC to explore the prognostic role of PC-related genes. Univariate Cox regression analysis (UCR) identified candidate genes that were significantly associated with OS (*p < 0.05).

In addition, the coefficients of many unrelated features were set to 0 according to the conditioning weight λ, and all regression coefficients were reduced to 0 by lasso. Subsequently, we analyzed a multivariate Cox regression (MCR) model to recognize pivot genes and calculate their corresponding coefficients. Finally, 11 pivot PC-related genes with prognostic risk models were established, and a risk score was computed [36].

All samples procured corresponding risk scores by risk formula. Each sample was divided into low-risk and high-risk subgroups when the median value of the risk score (0.9564) was set as the cut-off point. Firstly, the K-M survival curve was used employing the R package "survival" to identify prognostic differences. In addition, ROC curves were used to verify prognostic value. Subsequently, UCR and MCR were executed on the effectiveness of risk signature as independent prognostic features. R "pheatmap [37] " package was used to compare clinical features in low- and high-risk patients and visualize the correlation of risk score with clinicopathological variables [36].

### Establishment and identification of the nomogram

A ROC analysis was employed to identify the ideal prognostic indicator, risk score, gender, age, tumor grade and clinicopathological stage for 1/2/3-year OS [38]. In order to establish a quantitative prognostic prediction model for CC patients, we developed a nomogram combining risk score and other clinicopathological features to predict a 1/2/3-year OS rate. In addition, the calibration curve reflecting the predictive validity of the nomogram was constructed.

### Collection and pre-processing of epigenetic mutation data

TMB was considered to examine the number of base replacements, somatic, coding and insertion-deletion mutations per megabase of the genome at a 5% detection limit using the abbreviation for nonsynonymous and code-switched abbreviations [39]. The number of somatic nonsynonymous point mutations was calculated for each sample using the "maftools" R package [35].

### Correlation of risk score with TIME characterization

To define the relative between risk score and TIC, TIMER [18], XCELL, EPIC, CIBERSEORT-ABS, CIBERSORT, QUANTISEQ, and MCP counter were used to assess the immune environment. In addition, spearman correlation analysis was used to investigate the correlation between risk score and immune infiltration status and compare the differences in TIC scores between low and high-risk subgroups.

### Gene set variation analysis

Pathway analyses were constructed to assess the activation of characteristic pathways and metabolic pathways mentioned in the MSigDB databases (https://www.gsea-msigdb.org/gsea/msigdb). Additionally, to evaluate the correlation pathway activity in each sample, the GSVA package (version 1.36.3) was used to assign the path activity estimates [39].

### Prediction of patients’ response to immunotherapy

A total of 45 ICB-related genes were obtained, and their expression levels were investigated in low/high-risk samples. To further explore the underlying role of the risk score in immunotherapeutic prediction, IPS was taken as a determinant for quantifying tumor immunogenicity and featured the cancer antigenome and intratumoral immune landscape [40]. This scoring system was established based on effector cells, suppressor cells, checkpoints or immunomodulators (CP), and a weighted average Z-score. MHC-related molecules were calculated by averaging the Z-scores of samples from the four categories in their respective categories.

### Prediction of chemotherapeutic effect

R package pRRophetic was used to estimate IC50 of CC samples in various ICI score groups. The construction of regression models was done based on expression profiles of cancer drug sensitivity genomics (GDSC) (www.cancerrxgene.org/) cell lines and TCGA gene expression profiles [41].

### Experimental validation

A total of 10 clinical specimens of CC patients were obtained from the general surgery department of Jiangsu Province Hospital of Chinese Medicine for qRT-PCR testing A polyvinylidene difluoride membrane (Immobilon-P, Millipore, Billerica, MA, USA). Antibodies against the following proteins were following: *CD177* antibody ((Abmart Shanghai) 22-321AA), Anti-β-Tubulin (Abmart Shanghai M20005S), and Goat Anti-Rabbit Mouse IgG-HRP (Abmart Shanghai M21003). Blots were visualized using enhanced chemiluminescence reagents ECL (Biosharp BL523B China). In addition, we collected 10 clinical specimens of CC patients from the general surgery department of Jiangsu Province Hospital of Chinese Medicine for qRT-PCR (Quantitative Real-time Polymerase Chain Reaction) testing [42]. *β-actin* levels were used as the endogenous control, and the relative expression of *CD177* was calculated by the 2-ΔΔCt method. Primer sequences for PCR: *CD177*, 5′- TCATCTCTCAGGAGGTGGGC -3′ (forward) and 5′-CCAAGTGAGAGACTCCAGGC-3′ (reverse); *β-actin*, 5′- CCAACCGCGAGAAGATGA -3′(forward) and 5′- CCAGAGGCGTACAGGGATAG -3′(reverse).

### Statistical analysis

The Kruskal–Wallis test was used for comparisons between more than two groups, and the Wilcoxon test was used for comparisons between two groups. The Kaplan–Meier log-rank test was employed for survival curves. Relevant risk score subgroups with somatic mutation frequency were analyzed through the chi-square test, and the Spearman analysis was used to calculate the relation coefficient. For further analysis, the results of the CIBERSORT algorithm were *p* < 0.05. A *p*-value < 0.05 indicated statistical significance [42].

## Results

### Removing a batch effect

**Fig. 2:**
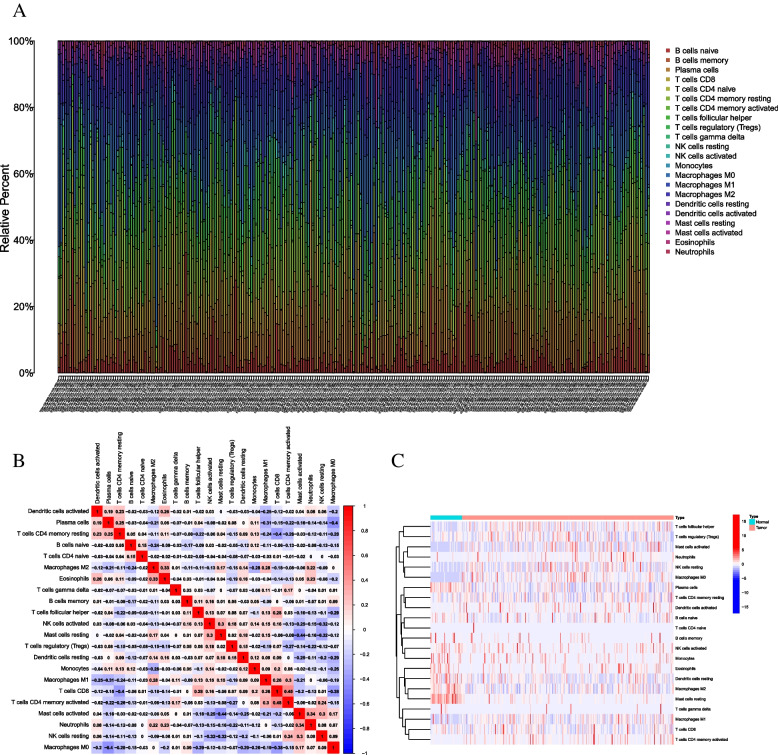
Landscape of immune cell infiltration in tumor immune environment of colon cancer. Subpopulation of 22 immune cell subtypes **A**. **B**. Intrinsic correlation of 22 infiltrating immune cells in colon cancer. C. Expression of subpopulation of 22 immune cell subtypes in tumor and normal tissues. The color from blue to red represents a trend of a negative correlation to a positive correlation

A total of 11,283 genes were obtained in two different CC cohorts (TCGA-CC Project). To identify the comprehensive landscape of TIME, the CIBERSORT algorithm was carried out (Supplementary file 1: Table S1). Figure 2A shows the abundance of 22 TIC types. To further reveal the underlying correlation between these TIC, the connection was employed to visualize the comprehensive landscape of TIME (Fig. 2B). PC was found to have the strongest negative correlation with PC (*p* < 0.05; *r* =  − 0.4), whereas PC was most positively correlated with T cells CD4 + memory resting (*p* < 0.05; *r* = 0.25).

### Establishment of the WGCNA network

**Fig. 3:**
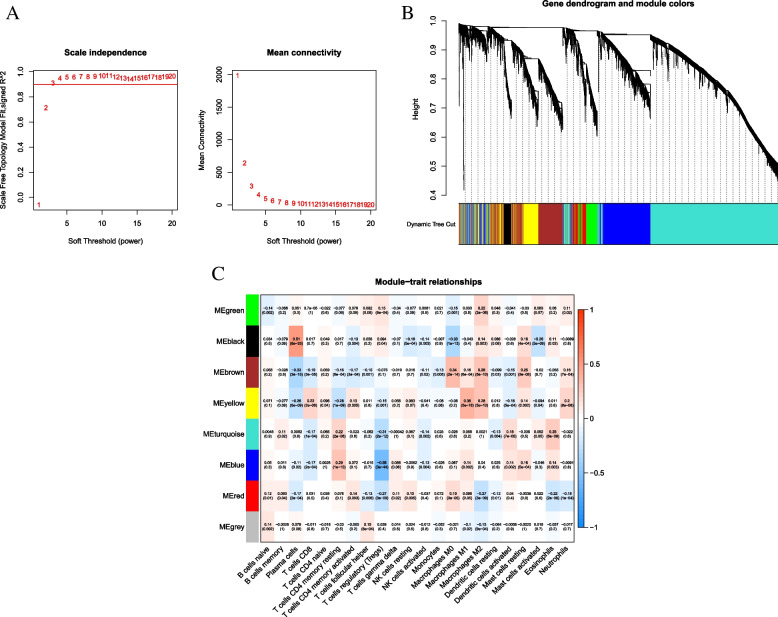
Selection of the appropriate soft threshold (power) and construction of the hierarchical clustering tree. **A** Selection of the soft threshold made the index of scale-free topologies reach 0.90 and analysis of the average connectivity of 1–20 soft threshold power. **B** Plasma cells-related genes with similar expression patterns were merged into the same module using a dynamic tree-cutting algorithm, creating a hierarchical clustering tree. **C** Heatmap of the correlations between the modules and immune-infiltrating cells (traits). Within every square, the number on the top refers to the coefficient between the cell infiltrating level and corresponding module, and the bottom is the *p* value

We performed immune infiltration subgroup analysis on sequencing files of 11,283 genes. The optimal soft threshold power (β) was set to 9 after establishing the scale-free network, as it was the first power value when the scale-free topology index reached 0.90 (Fig. 3A). The dynamic tree-cutting algorithm (module size = 8) introduced genes with similar expression patterns into the same module, enabling different modules to form hierarchical clustering trees. According to weighted correlation, hierarchical clustering analysis was employed, and the clustering outcomes were segmented based on the set criteria to acquire 8 gene modules (Fig. 3B). In Fig. 3C, each row shows the candidate module with characteristics vector genes, and each column presents the 8 TIC types. Among 8 candidate modules, the black module had the strongest correlation with PC (cor = 0.51, *p* = 5e-33). Therefore, PC-related genes from the black module (Supplementary file 1: Table S2) were used for further investigation.

### Development of risk signature

**Fig. 4:**
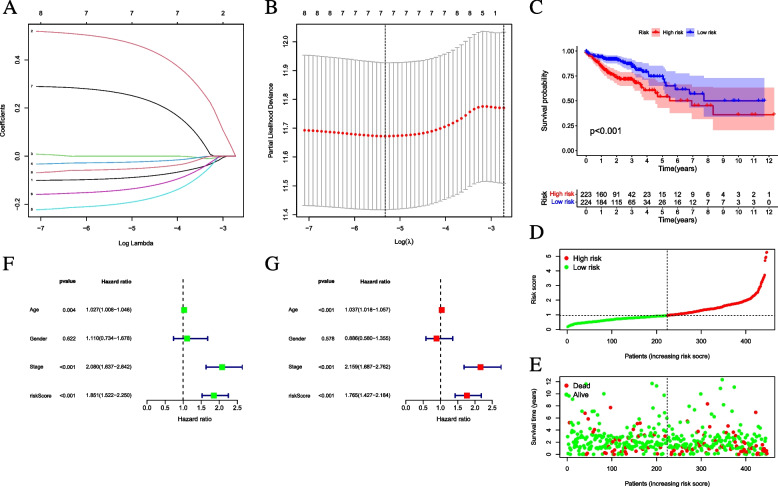
Establishment of the prognostic risk signature. **A** LASSO coefficient profiles of 71 candidate genes. A vertical line is drawn at the value chosen by tenfold cross‐validation. **B** Ten‐time cross‐validation for tuning parameter selection in the lasso regression. The vertical lines are plotted based on the optimal data according to the minimum criteria and 1-standard error criterion. The left vertical line represents the 5 genes finally identified. **C** Kaplan–Meier curve analysis presenting difference of overall survival between the high-risk and low-risk groups. **D** Distribution of multi-genes model risk score. **E** The survival status and duration of CC patients. **F** Univariate Cox regression results of overall survival. **G** Multivariate Cox regression results of overall survival

**Fig. 5:**
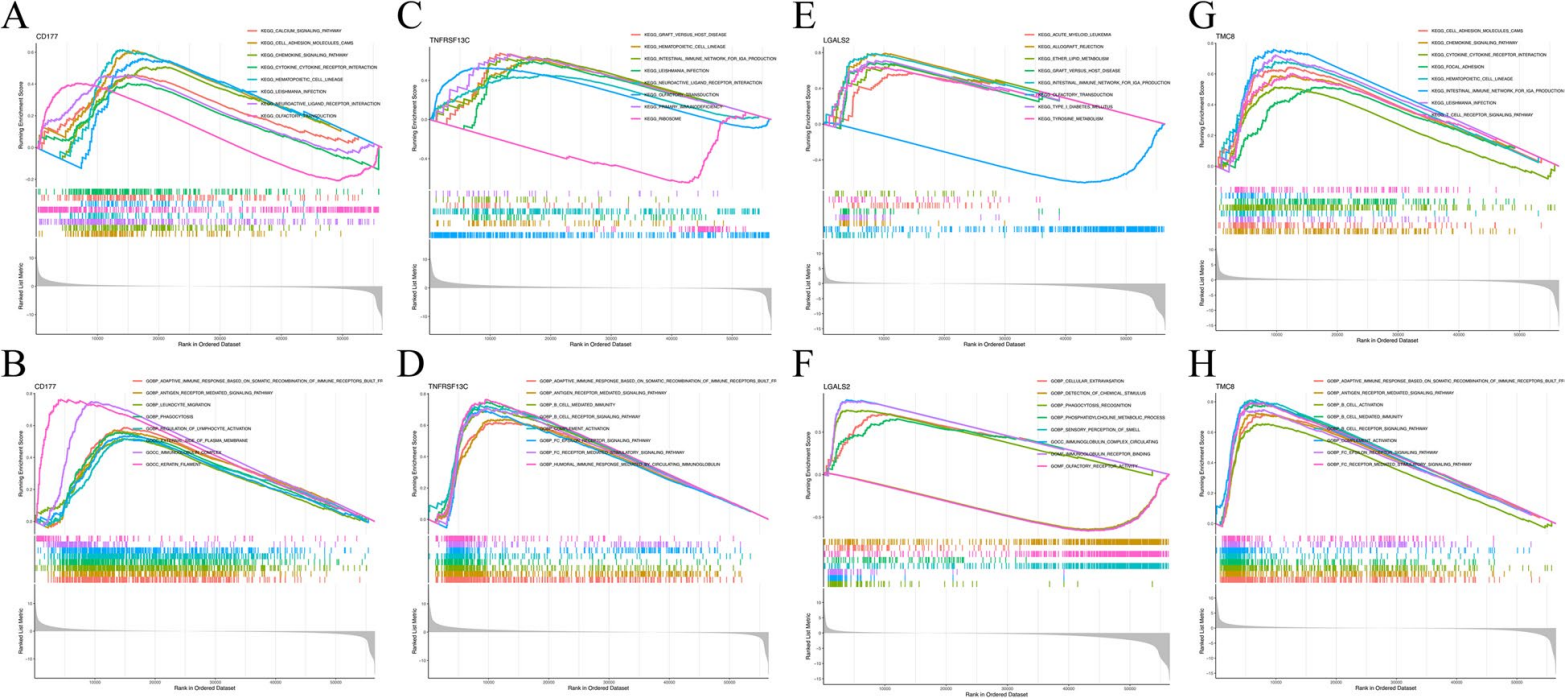
GSEA for samples with high and low expression of 4 hub genes. **A** The enriched gene sets in KEGG collection by the high *CD177* expression sample. **B** The enriched gene sets in GO collection by the high *CD177* expression sample. **C** The enriched gene sets in KEGG collection by the high *TNFRSF13C* expression sample. **D**. The enriched gene sets in GO collection by the high *TNFRSF13C* expression sample. **E** The enriched gene sets in KEGG collection by the high *LGALS2* expression sample. **F** The enriched gene sets in GO collection by the high *LGALS2* expression sample. **G**. The enriched gene sets in KEGG collection by the high *TMC8* expression sample. **H** The enriched gene sets in GO collection by the high *TMC8* expression sample

The expression data and follow-up information from the TCGA-CC project were obtained to further investigate the prognostic value of candidate genes. Eight PC-related genes were determined with importance prognostic value by UCR (*p* < 0.05, Supplementary 1: Table S4). Prognostic features were performed for these hub genes; lasso regression was employed to avoid overfitting. Finally, 5 PC-related genes correlated with CC prognosis were identified (Fig. 4A). After 10 rounds of cross-validation, the optimal value of the penalty parameter was established (Fig. 4B). After MCR analysis, five PC-related genes, i.e., *CD177*, *LGALS2*, *TMC8*, *TNFRSF13C*, and *VWA5A,* were identified, and all were regarded as prognostic indicators (*p* < 0.05, Supplementary file 1: Table S5). High expression of 3 hub gene (*VWA5A*, *CD177*, and *LGALS2*) was positively correlated with prognosis. *TMC8* and *TNFRSF13C* were the opposite, as shown in Fig. 5.

The genome in the TCGA database demonstrated significantly different expression patterns in CC tissues compared to normal tissues (Supplementary file 2: Figure S1A–E). The HPA database showed that proteins (*CD177*, *LGALS2*, *TMC8*, and *VWA5A*) were significantly dysregulated in tumor tissue relative to normal samples (Supplementary file 2: Figure S2A–J). In addition, survival analysis of most hub genes showed abnormal mRNA expression that resulted in significantly different OS times (most *p* < 0.05, Supplementary file 2: Figure S3A–F).

All samples were divided into high/low expression groups based on the median expression of the hub gene. Subsequently, GSEA identification function enrichment was conducted on the high/low-expressed hub gene (Supplementary file 1: Table S3).

As shown in Fig. 5A, KEGG revealed that high expression of *CD177* was concentrated in the cell adhesion molecule signaling pathway, neuroactive ligand-receptor interaction signaling pathway, and calcium signaling pathway. Genesets uncovered that high *CD177* expression was mainly associated with keratin filament, immunoglobulin complex, and regulation of lymphocyte activation (Fig. 5B). As shown in Fig. 5C, the three KEGG demonstrated high expression of TNFRSF13, which was positively enriched in the primary immunodeficiency signaling pathway, graft versus host disease signaling pathway, and neuroactive ligand-receptor interaction signaling pathway. Figure 5D demonstrates that the GO pathway had the most significant correlation with high TNFRSF13 expression. The high expression with TNFRSF13 was mainly in adaptive immune response based on somatic recombination of immune, B cell receptor signaling pathway, antigen receptor-mediated signaling pathway, and B cell-mediated immunity. KEGG showed that the *LGALS2* had high expression in the allograft rejection signaling pathway, acute myeloid leukemia, and intestinal immune network for IgA production (Fig. 5E). Moreover, the GO pathways in complex immunoglobulin circulating and immunoglobulin receptor binding were identified as the most *LGALS2*-relevant signaling pathways (Fig. 5F). KEGG enrichment term revealed that the high expression of *TMC8* was mainly associated with the intestinal immune network for IgA production (Fig. 5G). Figure 5H shows that the GO pathway was most significantly correlated with high *TMC8* expression. The high expression of *TMC8* was mainly in B cell receptor signaling pathways. However, *VWA5A* did not enrich the relevant signaling pathways and the GO terms. In addition, we calculated risk scores for 5 hub genes in the risk profile of CC patients: risk score = (− 0.1453 ∗ expression of *CD177*) + (− 0.2687 ∗ expression of *VWA5A*) + (− 0.1755 ∗ expression of *LGALS2*) + (− 0.5049 ∗ expression of *TMC8*) + (− 0.2988 ∗ expression of *TNFRSF13C*). Finally, the corresponding risk score was classified into low-risk and high-risk subgroups based on the median cut-off value of the CC samples (1.3001).

### Validation of risk prognostic signature

K-M survival curves showed significantly lower OS times in high-risk samples than in low-risk samples (*p* < 0.001; Fig. 4C). Moreover, distributions of the dot pot of survival status and risk score indicated shorter OS for high-risk CC patients (Fig. 4D, E). Next, UCR showed the hazard ratio (HR) of the risk score of 1.851 (95% CI 1.522 − 2.250; Fig. 3F). Finally, the results of MCR (HR = 1.765, 95% CI 1.427 − 2.184; Fig. 3G) pointed to risk score as an independent prognostic indicator for CC. These outcomes demonstrated that these five hub gene features could predict clinical prognosis.

### Correlation of risk signature with clinicopathological variables

**Fig. 6:**
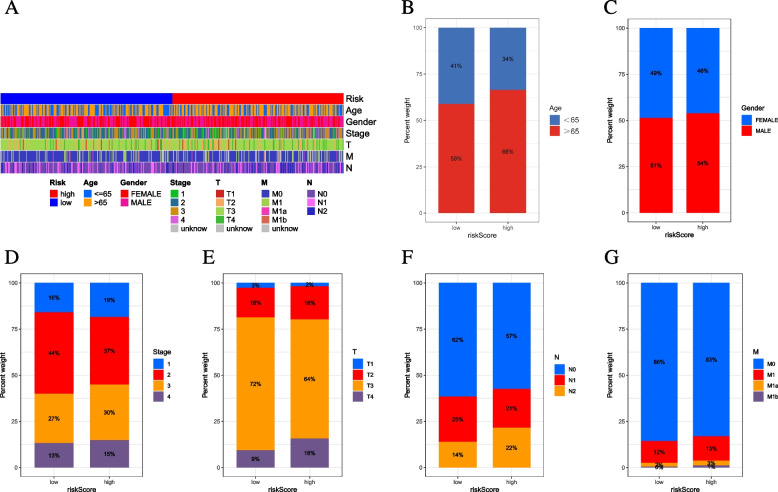
Clinical significance of the prognostic risk signature. **A** Heatmap presents the distribution of clinical feature and corresponding risk score in each sample. Rate of clinical variables subtypes in high or low risk score groups. **B** Age, **C** Gender, **D** clinical stage, **E** T status, **F** N statusand **G** M status

As shown in Fig. 6A, the distribution of clinical variables in the high/low-risk subgroups was recognized and visualized. Figures 6B-G show the proportion of clinical subtypes based on age, gender, clinical stage, tumor grade, N category and T status in the low/high-risk subgroups.

### Construction of prognostic nomogram

**Fig. 7:**
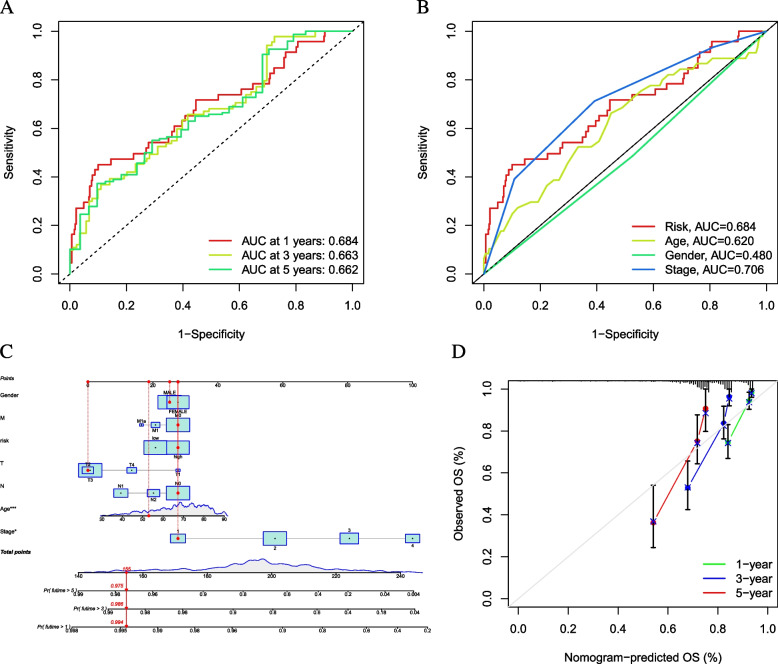
Validation of prognostic efficiency of risk signature. **A** ROC analysis was employed to estimate the prediction value of the prognostic signature. **B** Areas under curves (AUCs) of the risk scores for predicting 1-, 3-, and 5-year overall survival time with other clinical characteristics. **C** Nomogram was assembled by stage and risk signature for predicting survival of CC patients. **D** One-3–5-year nomogram calibration curves

As shown in Fig. 7A, ROC curves were plotted with AUC values of 0.684, 0.663, and 0.662 for 1-, 3-, and 5-year OS, demonstrating excellent prognostic ability. To further demonstrate that risk score is the most effective prognostic indicator among multiple clinicopathological variables, we designated age, gender, and clinical stage as candidate prognostic factors.

These clinical characteristics were included in the AUC analysis for 1-year, 3-year, and 5-year OS, and we found that clinical staging acquired the highest AUC values (Fig. 7B). Subsequently, a prognostic nomogram, including risk score and clinical staging, was used to predict prognosis (Fig. 7C) quantitatively. Finally, calibrate curves demonstrated that the nomogram model had excellent prognosis predictive performance (AUC value > 0.5, Fig. 7D).

### Correlation of risk signature with TMB

**Fig. 8:**
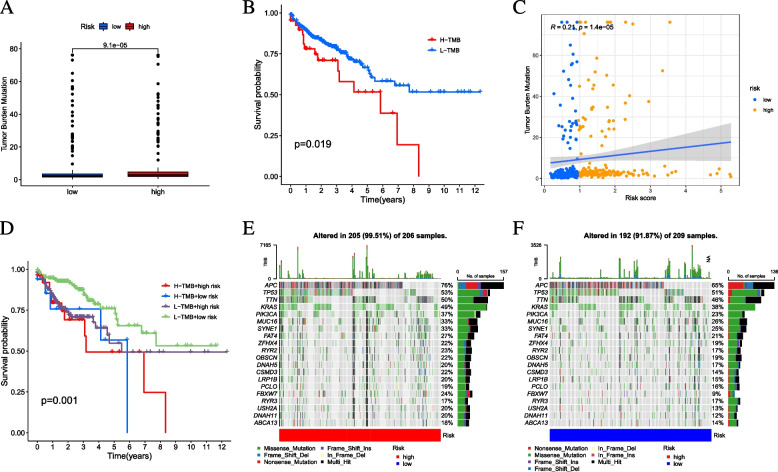
Validation of prognostic efficiency of risk signature. **A** ROC analysis was employed to estimate the prediction value of the prognostic signature. **B** Areas under curves (AUCs) of the risk scores for predicting 1-, 3-, and 5-year overall survival time with other clinical characteristics. **C** Nomogram was assembled by stage and risk signature for predicting survival of CC patients. **D** One-3–5-year nomogram calibration curves

First, TMB levels were detected in both high- and low-risk score subgroups. Our results revealed that the low-risk score subgroup had lower TMB levels than the low–high-risk sample (*p* = 9.1e-05, Fig. 8A). Patients were assigned to different subtypes according to the TMB immune set point [43]. Survival curves showed that the low TMB values had longer OS time (*p* = 0.019, Fig. 8B). A correlation analysis further validated the positive association between TMB and risk score (R = 0.21, *p* = 1.41–05; Fig. 8C). Subsequently, we validated the combined effect of risk score and TMB in the prognosis prediction of CC. Stratified survival curves for risk score subgroups in low and high TMB status subtypes showed significant prognostic differences (*p* = 0.001; Fig. 8D). In sum, the results demonstrated that risk score might serve as an independent prognostic predictor to assess the clinical prognostic of anti-tumor immunotherapy.

In addition, the distribution among high-risk and low-risk scoring subtypes in gene mutations was also identified and visualized. The mutation patterns and clinical characteristics of the top 20 most frequently altered driver genes are shown in Fig. 8E, F. The mutation landscape revealed that APC (76% vs. 65%) had a higher somatic mutation rate in the high-risk score subtype, while ABCA13 (18% versus 14%) had a higher somatic mutation rate in the low-risk score subgroup. The above results provide insight into the intrinsic link between somatic cell mutations and plasma infiltration in CC immunotherapy.

### Risk characteristics in the TIME context of CC

**Fig. 9:**
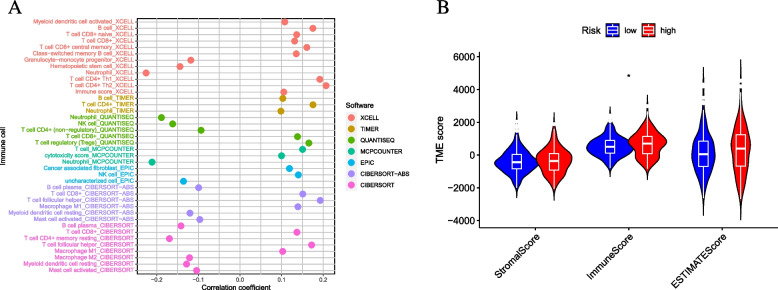
Estimation of abundance of tumor-infiltrating cells. **A** Patients in the high-risk group were more positively associated with tumor-infiltrating immune cells, as shown by Spearman correlation analysis. **B** Correlation between prognostic risk signature with hub immune checkpoint genes

The intrinsic connection between PC-based risk score and TIC explores the fundamental contribution of a risk score to the sophisticated variety of TIME. The outcomes demonstrated that risk score was negatively correlated with subpopulations of resting T cell CD4 + memory cells, B cells, macrophage M2, resting myeloid dendritic cells while positively associated with plenty of macrophage M1, B PC, T cell follicular helper, T cell CD4 + Th1, Tregs, T cell CD4 + Th2, CD8 + (Supplementary file 2: Figure S4). In addition, as shown in Fig. 9A, we further analyzed the Spearman association of risk score with immune infiltration; the detailed results are presented in Supplementary file 1: Table S6.

There was a significant upward trend in stromal scores and immune scores in the low-risk group and a significant upregulation of ESTIMATE scores in the low-risk samples (Fig. 9B).

### Enrichment of signaling pathways in high/low-risk groups

**Fig. 10:**
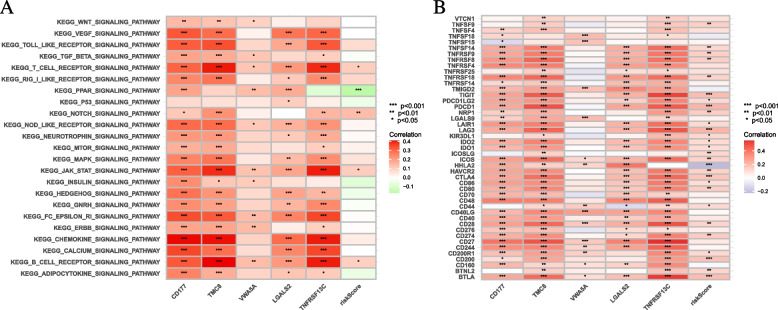
Enrichment pathways of GSVA. **A** Heatmap showing the correlation of representative pathway terms of KEGG with risk score. Prediction of Immunotherapeutic Response. **B** Correlation of expression level of immune checkpoint blockade genes with risk score

GSVA was used to explore the biological role of different risk groups in tumorigenesis and progression (Fig. 10A). Enhanced activity of the CHEMOKINE pathway, JAK/STAT pathway, T-cell receptor signaling pathway, and B-cells receptor pathway were found in the low-risk group. Gene with high expression levels was enriched in the P53 pathway, INSULIN pathway and PPAR pathway in high-risk groups.

### Predicting patients’ clinical outcome to immunotherapy

Next, the response to immunotherapy was further explored. Most of the genes associated with ICB had important positive associations with risk score (Fig. 10B), and the genes with strong correlations were PDCD1, CD40LG, CD28, CD27, and BTLA. However, the risk scoring system revealed that scores for IPS-PD1 and CTLA-4 blockers did not significantly differ (Supplementary file 2: Figure S5). These results suggested that risk score was potentially associated with the response to immunotherapies.

### Prediction of response to chemotherapy

The IC50 of 23 chemotherapeutic medicines was estimated in CC patients according to the pRRophetic algorithm. These chemotherapeutics revealed higher IC50 in lower-risk patients (*p* < 0.05; Supplementary file 2: Figure S6), thus suggesting that chemotherapeutic agents are more effective in low-risk samples.

### Differential expression of *CD177* in samples and cells

We performed qRT-PCR validation on five genes and found that the expression of the *CD177* gene in samples of tumor and normal tissues was more significant and stable than the other four genes (Supplementary file 2: Figure S8A-E). Combined with previous literature studies, we further validated and analyzed CD177 [23, 24, 44–46].

**Fig. 11:**
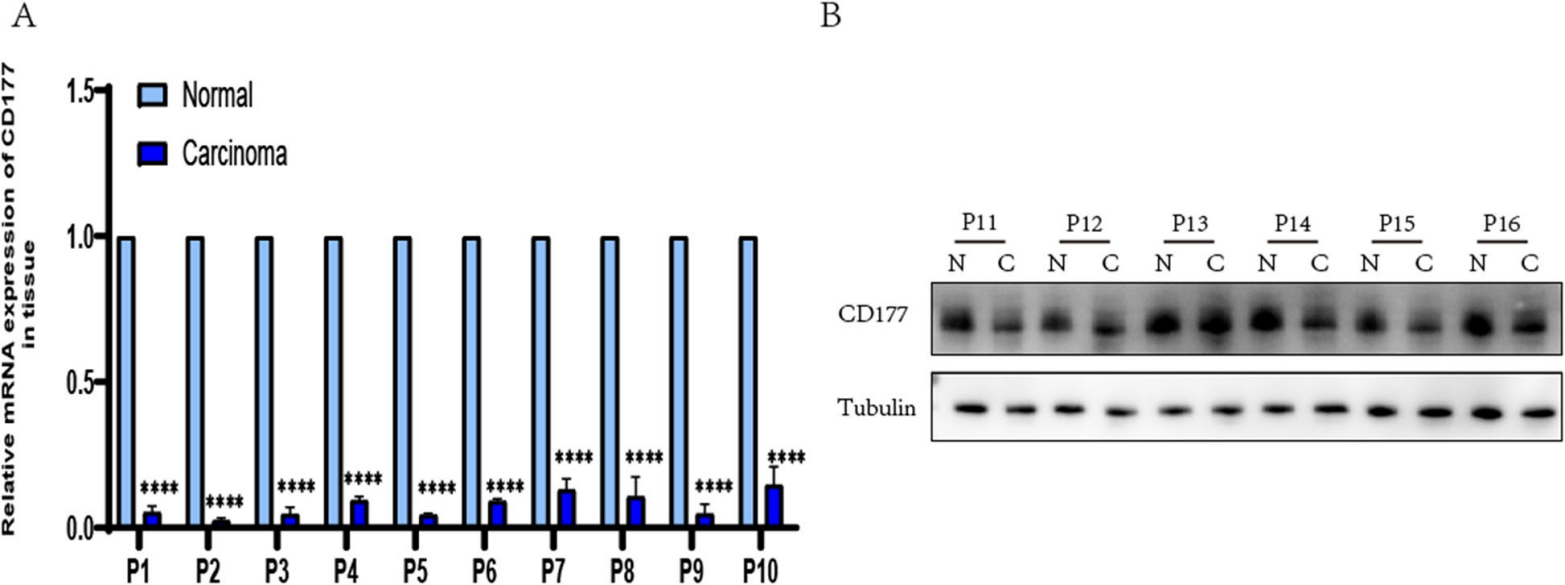
Expression pattern of *CD177* in human colon cancer. **A** qRT-PCR of *CD177* expression in 10 pairs of CC tissues and adjacent nontumour tissues. **B** Western blot of the protein levels of *CD177* in normal tissue and cancer tissue

Dysregulated expression levels of *CD177* were most pronounced in these prognostic plasma cell-associated genes. Moreover, the biological function of the *CD177* gene in CC was further explored in a subsequent trial. As shown in Fig. 11A, we examined 10 pairs of clinical samples by qRT-PCR, finding that *CD177* expression in CC was substantially lower than in the adjacent normal tissue. In addition, low *CD177* expression was observed in six patient cancer tissues from the protein level, while normal tissues had relatively high *CD177* expression (Fig. 11B). This indicated more *CD177* infiltration in normal tissue samples, suggesting *CD177* as a potential target for CC.

## Discussion

Due to the metastasis and recurrence of tumors, CC has become one of the diseases with a high mortality rate [47]. It is well known that gene mutations, genomic variants, and the regulation of non-coding RNA [48] and EMT [49] are key regulators of CC progression. With the development of immunotherapy, immune checkpoint immunotherapy has become an important tool for anti-cancer treatment [50–52]. Compared to most other therapies for metastatic tumors, immunotherapy has achieved long-term durable remissions in a subset of patients, with promising prospects in treating dMMR–MSI-H metastatic CC [53].

In the investigation of CC, TIC has been gaining increasing importance [54, 55]. Overwhelming evidence demonstrated that TIC contributes positively to anti-tumor immunity. CD138 (syndecan-1) has become the most commonly utilized marker for assessing PC infiltration. In addition, PC has shown that the constant structure of the IGKC gene encoding the immunoglobulin kappa light chain is highly expressed. Thus, compared to CD138, IGKC might be more suitable for detecting PC. Several previous studies have demonstrated that IGKC has a positive prognostic effect on CC [56]. Furthermore, PC produces tumor-specific antibodies that bind to tumor cells, inhibiting their target proteins, activating complement, and promoting antibody-dependent cytotoxicity. UCR, lasso, and MCR were performed to identify five hub genes, after which the risk score was calculated, and prognostic markers were constructed. K-M analysis and ROC curves validated the good predictive performance of the risk model. We revealed that the risk characteristics could be a good independent prognostic predictor in UCR and MCR. In addition, risk characteristics remained strong prognostic factors in the stratified survival curves of clinical variables. The above results suggest that five genetic risk markers can be used as independent prognostic molecular biomarkers for predicting clinical outcomes in CC. In addition, we constructed and validated a prognostic risk score-age nomogram to provide a basis for clinical practice.

*CD177* is a glycosylphosphatidylinositol-anchored glycoprotein expressed in neutrophils [57]. Zhou et al*.* found that highly increased *CD177* expression in CC and UC and a higher density of *CD177* + neutrophils in CC predicted a better prognosis for CC patients. In addition, *CD177* deficiency promotes the inflammatory response, proliferation, and tissue remodeling of colonic epithelial cells, which might enlarge tumor size and increase tumor formation in *CD177*-/- mice [58]. Several studies reported that the *CD177* gene is a candidate gene that can predict a good prognosis for CC [59, 60]. Takeshi et al*.* suggested that the upregulation of *CD177* in gastric cancer (GC) also predicted a favorable prognosis for these patients. In addition, multivariate analysis revealed that high *CD177* expression in GC could be an independent prognostic condition for OS [45]. The biological role of *CD177* in tumors was investigated using a GSEA enrichment assay, revealing that *CD177* with high expression was mainly enriched in keratin filament, immunoglobulin complex, and regulation of lymphocyte activation. These results suggest that *CD177* is extensively involved in regulating tumor immune signaling pathways, thus further elucidating the role of *CD177* in anti-tumor strategies on a computational and bioinformatic basis.

Some clinical data point to an association between genetic alterations and responsiveness to immunotherapy [61, 62]. TMB, a predictor of immunotherapy sensitivity, increased significantly with an increasing risk score. In this work, the rate of ABCA13 mutations was significantly increased in the low-risk score subtypes, while the rate of mutations in SMGs of APC was increased in high-risk score patients. Research shows that mutation of APC conserved domain results in binding one of the oligomeric structural domains to IQ-motif-containing GTPase activation protein 1 (IQGAP1), PP2A, Asef, and KAP3 [63–66]. These interactions largely stimulate cell migration and cell adhesion, thus promoting tumor metastasis.

We further investigated the biological function of risk score in TIME characterization and immunotherapy. Our results demonstrated that risk score was positively associated with activated CD4 + T cells, B cells, and neutrophils, thus suggesting that the immune activation phenotype of the high-risk subgroup matches the OS dominance. Furthermore, the enrichment of higher stromal scores in the high-risk group suggested that stromal elements were activated, which might inhibit the anti-tumor effects of immune cells. GSVA results indicated that the high-risk group was associated with the P53 signaling pathway and NOTCH signaling pathway, while JAK/STAT signaling pathway, MAPK signaling pathway, and mTOR signaling pathway were activated in the low-risk group. These results showed a diversity of potential molecular mechanisms among the different risk samples.

However, IPS-PD1 and CTLA-4 blockers did not significantly differ in the risk score system scores. A previous study of the clinical response of PD1 to the treatment of tumors, melanoma, renal-cell cancer, and non–small-cell lung cancer presented a pronounced objective response, whereas no objective responses were observed in patients with prostate cancer or CC [67]. Another research demonstrated that mismatch repair-deficient, locally advanced rectal cancer was highly sensitive to single-agent PD-1 blockade, but prolonged follow-up is required to evaluate the duration of response [63]. In the present study, risk score showed a significant positive association with ICB-related genes, suggesting that high-risk samples were more closely associated with ICB. These current measures may have limitations in applicability and should be further investigated.

The present study further elucidated the impact of the prognostic properties of *CD177* on TIME characteristics and immunotherapy. First, our results showed that *CD177* had significantly low expression in CC samples, thus indicating it could serve as a poor prognostic predictor in CC. Thus, finding an immune-related biomarker to indicate the prognosis of CC is of utmost importance. Other studies based on TCGAs analyzed many targets, such as CXCL11, CADM3, LEP, CD1B, etc. [68, 69], and the advantages of targets from different aspects. However, in our study, *CD177* mRNA levels in the TCGA database had more significant differences in mean expression in CC samples and normal tissue samples, and the immunohistochemical differences were significantly better compared with other genes. Secondly, the differential expression and mutational burden of the *CD177* gene had a more prominent effect on the prognosis of tumor patients. In addition, we performed several experiments to verify *CD177* expression in CC, including mRNA levels and protein levels, all of which indicated that *CD177* might be an important target of interest. To the best of our knowledge, this is the first study that reported *CD177* in CC. This conclusion is expected to provide new targets and directions for the immunotherapy of CC in the future. In summary, the expression pattern of the *CD177* gene may be a promising target for CC therapy.

In conclusion, this study deciphered the TIME landscape through different datasets and comprehensive bioinformatics analysis. In addition, PC-based risk score schemes supported TIME heterogeneity, mutations, clinical prognosis prediction, and therapeutic response. Furthermore, the potential role of *CD177* in CC was further elucidated.

The interaction between CC and its tumor microenvironment seriously affects tumor evolution, affecting subtype classification, recurrence, drug resistance, and overall prognosis of patients. Although previous reports have provided elegant analysis of how the activation of intrinsic genes in tumors shapes the tumor microenvironment [70], we assessed genes that characterize the tumor microenvironment, eventually influencing the development of CC and thus contributing to the overall survival of patients. Our results may provide additional data for decoding the complex interactions of tumors with the tumor environment in CC.

However, due to the pandemic, follow-up data for CC patients are currently lacking, and we cannot conduct more experimental studies on the other four key genes. We hope to add these analyses to future work in order to collect more clinical data and perform more molecular experiments that could further validate reported findings.

## Supplementary Information

**Additional file 1.** 

**Additional file 2.** 

## Data Availability

The datasets generated and analyzed during the current study are available in the TCGA (https://portal.gdc.cancer.gov/cart). And, within the supplemental material (Additional files 2) provided with this article. Reference pictures were created using The Human Protein Atlas (https://www.proteinatlas.org/ENSG00000204936-CD177/tissue and https://www.proteinatlas.org/ENSG00000204936-CD177/pathology).

## Abbreviations

TIME: Tumor immune microenvironment
CC: Colon cancer
IMI: Immune cell infiltration
PC: Plasma cells
WGCNA: Weighted gene co-expression network analysis
ROC: Receiver operating characteristic
GSVA: Gene set variation analysis
IPS: Immunophenoscore
IC50: Half-maximal inhibitory concentration
TMB: Tumor mutation burden
TCGA: The Cancer Genome Atlas
GO: Gene ontology
KEGG: Kyoto Encyclopedia of Genes and Genomes
ESTIMATE: The MAlignant Tumors using Expression data
TC: Treg cells
OS: Overall survival
ICB: Immune checkpoint blockade
TIC: Tumor-infiltrating immune cells
UCR: Univariate Cox regression analysis
MCR: Multivariate Cox regression
CP: Checkpoints immunomodulatorisk score
GDSC: Genomics of Drug Sensitivity in Cancer
