# Supplementary figures and images for: Clinical plasma cells-related genes to aid therapy in colon cancer
Source: BMC Genomics. 2023 Aug 1;24:430. doi: 10.1186/s12864-023-09481-4 (PMC10391883; doi:10.1186/s12864-023-09481-4)

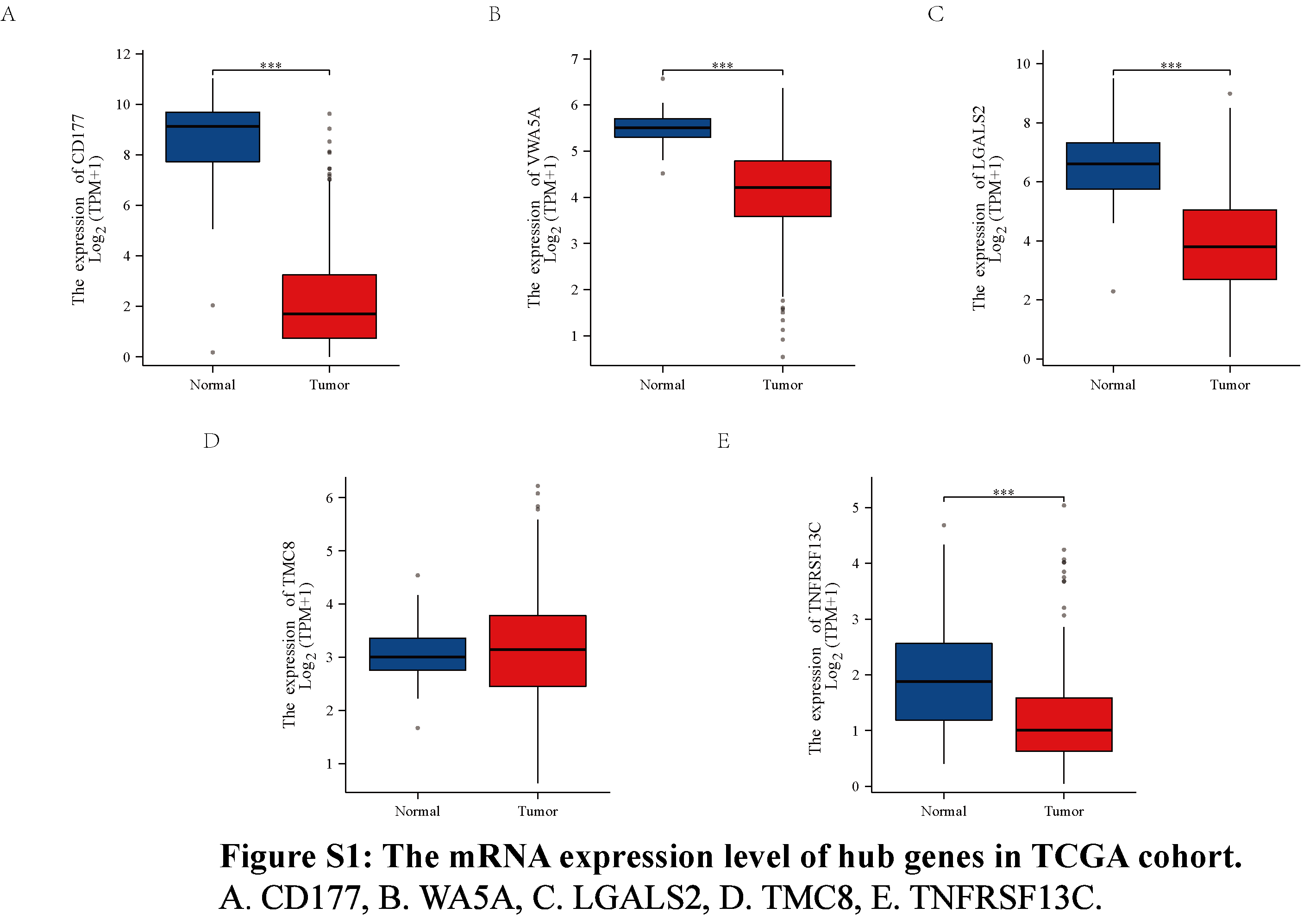

Supplement: Supplementary file 2 — Additional file 2. [file 12864_2023_9481_MOESM2_ESM.zip › Figure S1.tif]

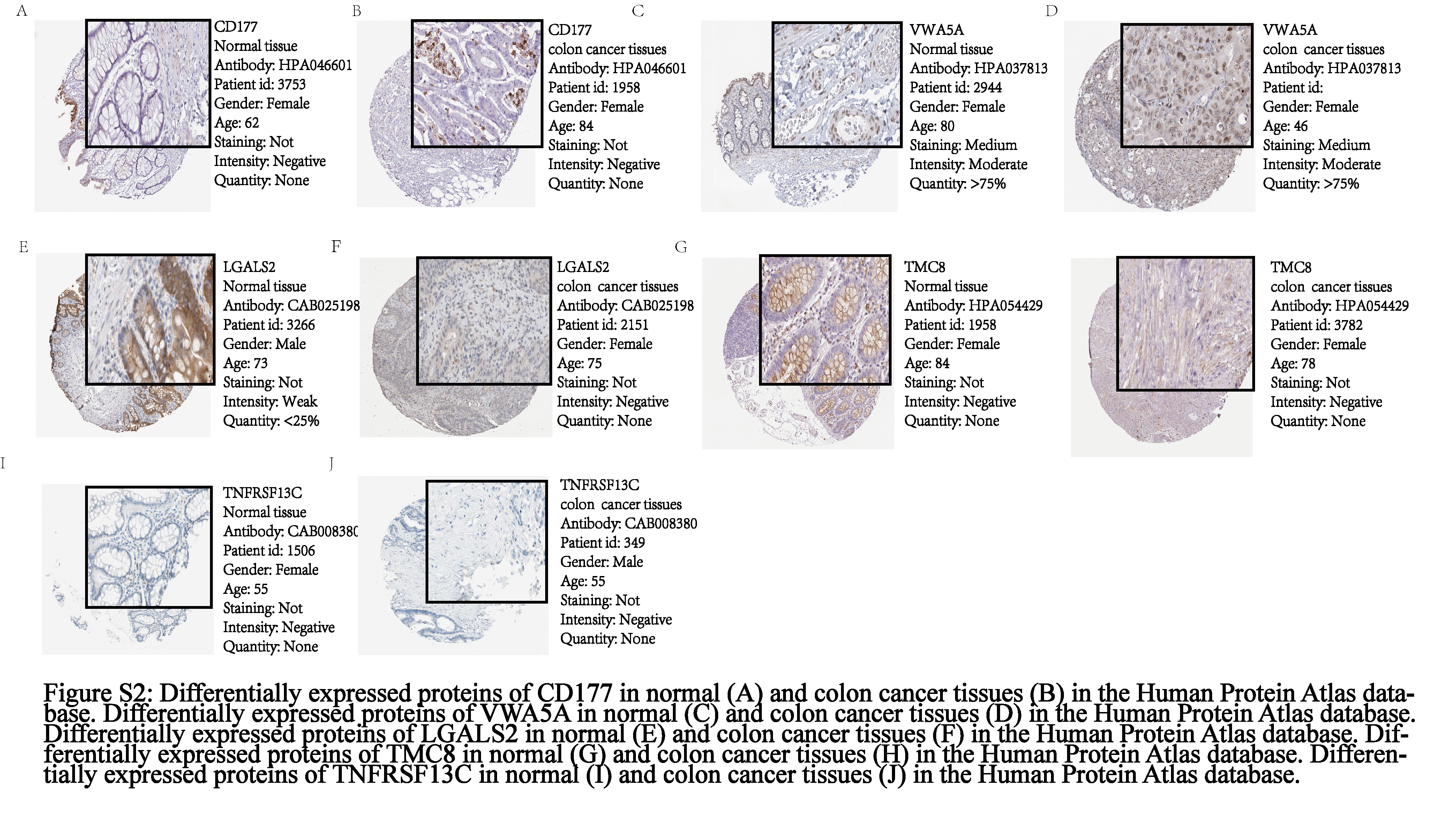

Supplement: Supplementary file 2 — Additional file 2. [file 12864_2023_9481_MOESM2_ESM.zip › Figure S2.tif]

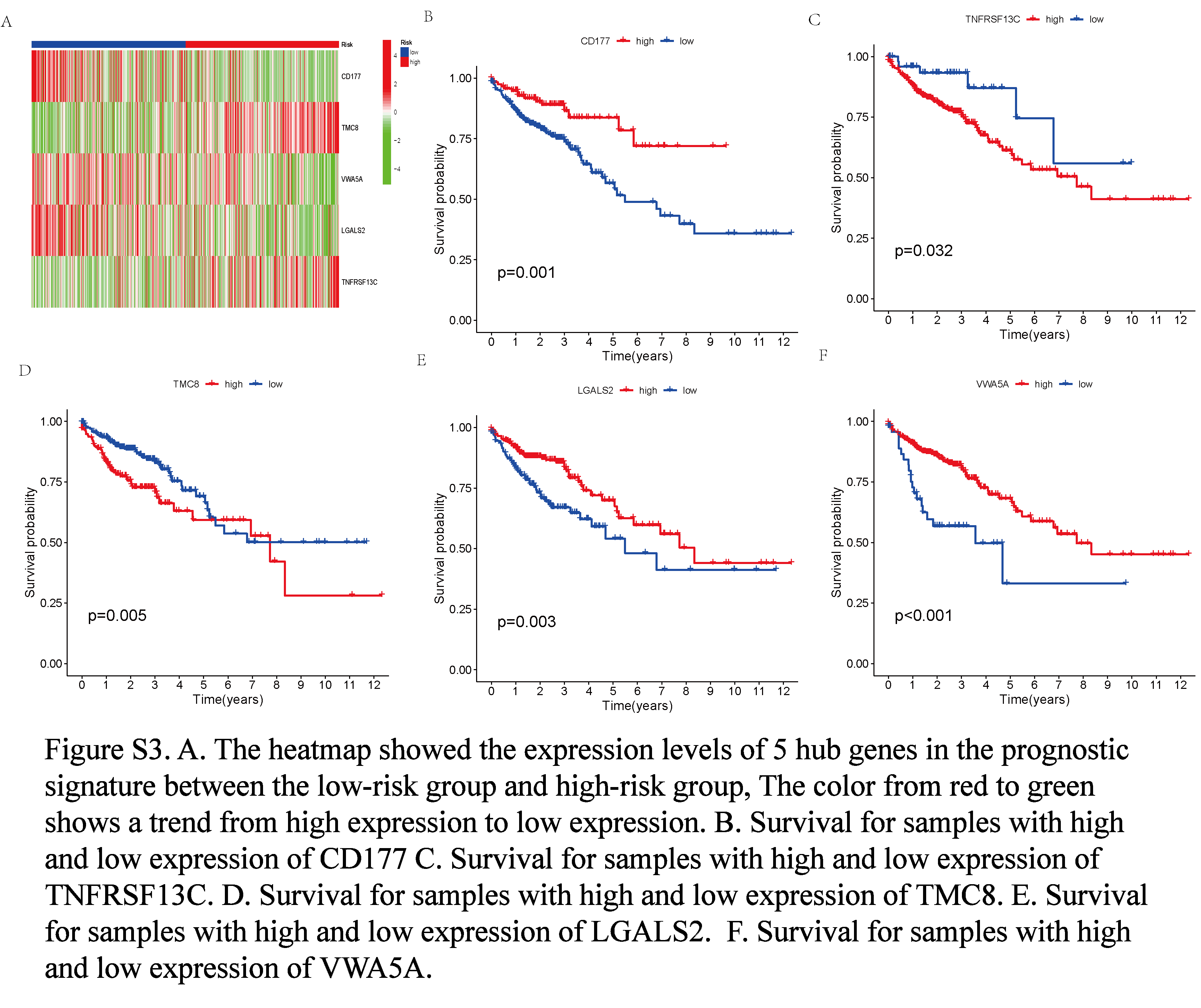

Supplement: Supplementary file 2 — Additional file 2. [file 12864_2023_9481_MOESM2_ESM.zip › Figure S3.tif]

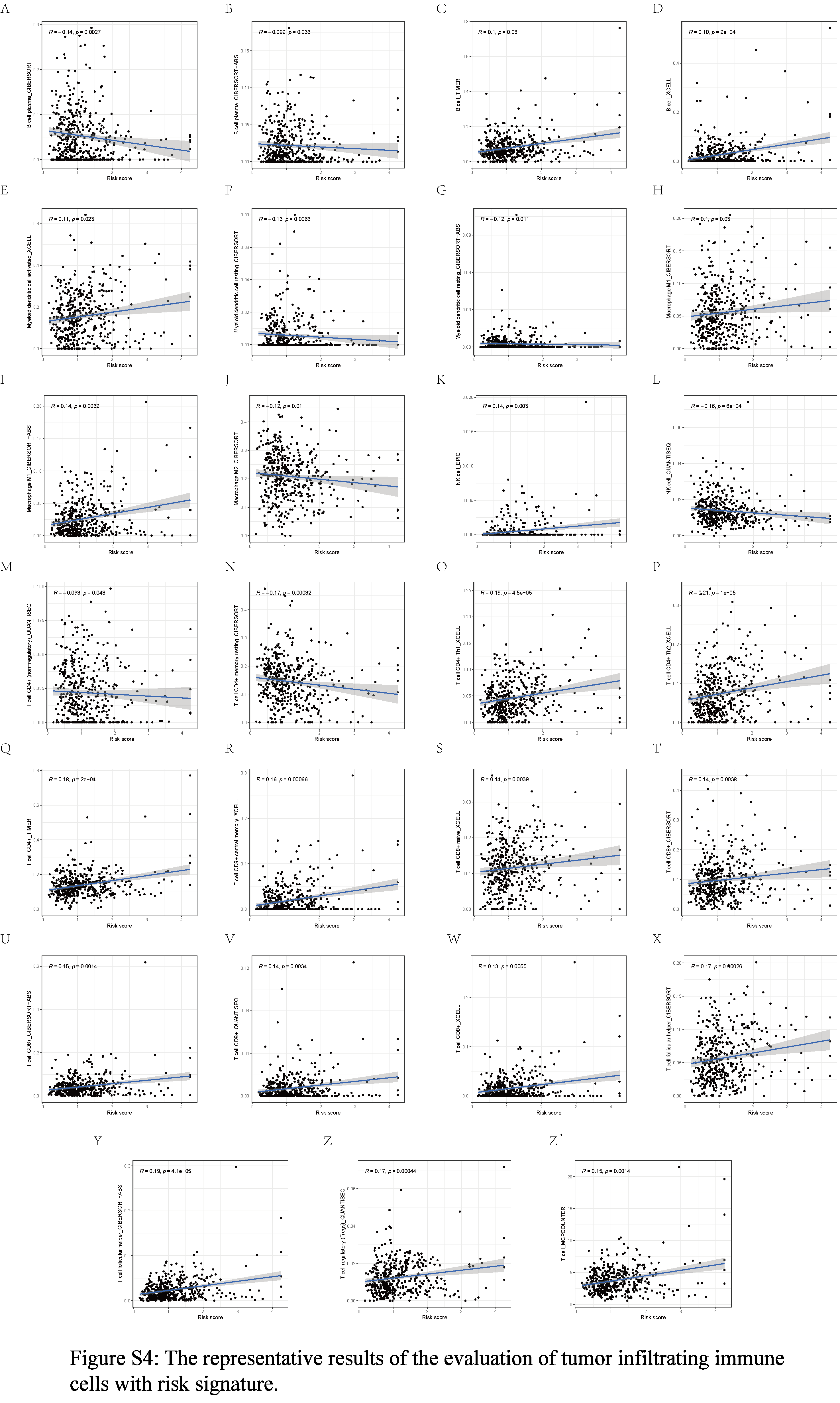

Supplement: Supplementary file 2 — Additional file 2. [file 12864_2023_9481_MOESM2_ESM.zip › Figure S4.tif]

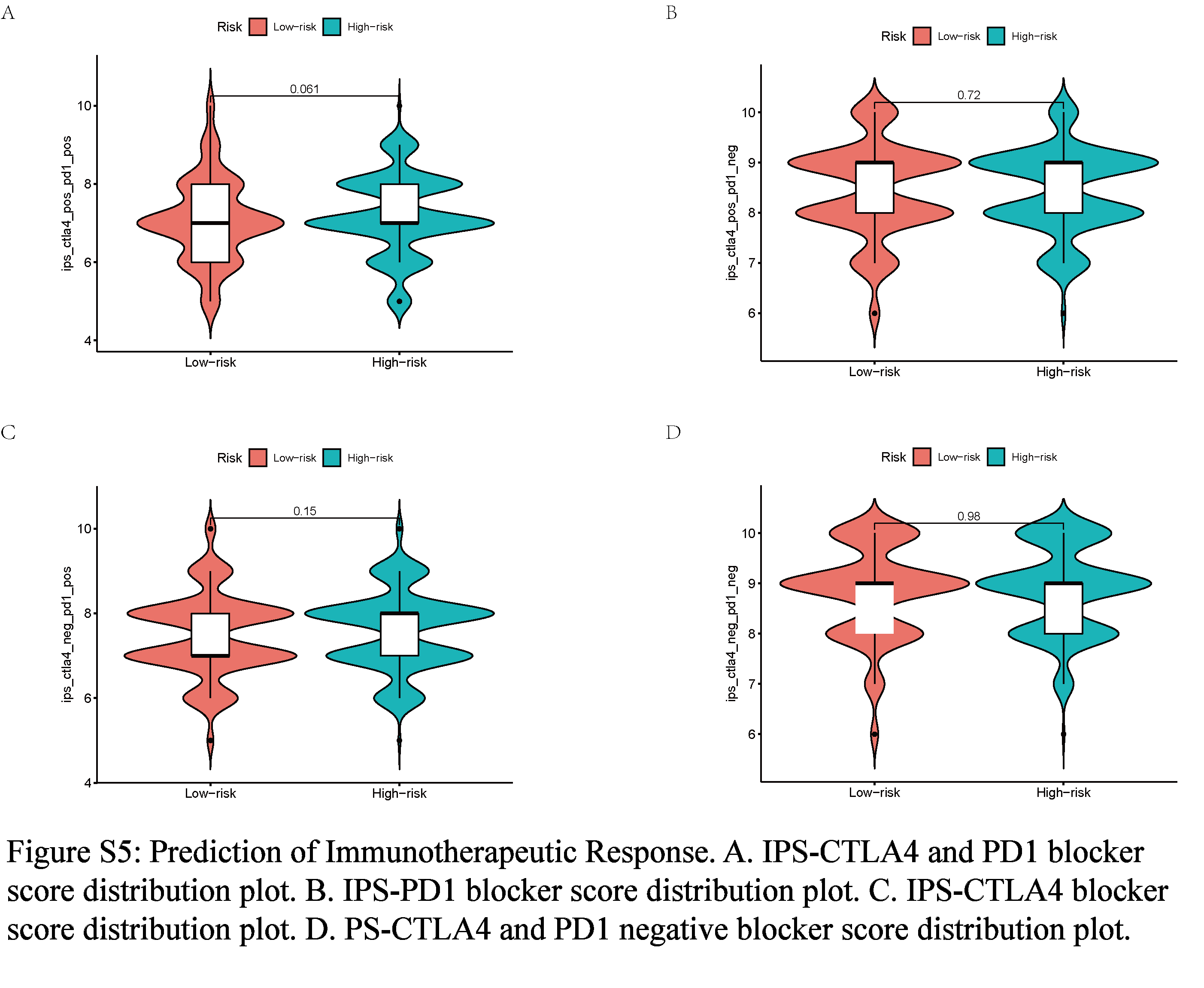

Supplement: Supplementary file 2 — Additional file 2. [file 12864_2023_9481_MOESM2_ESM.zip › Figure S5.tif]

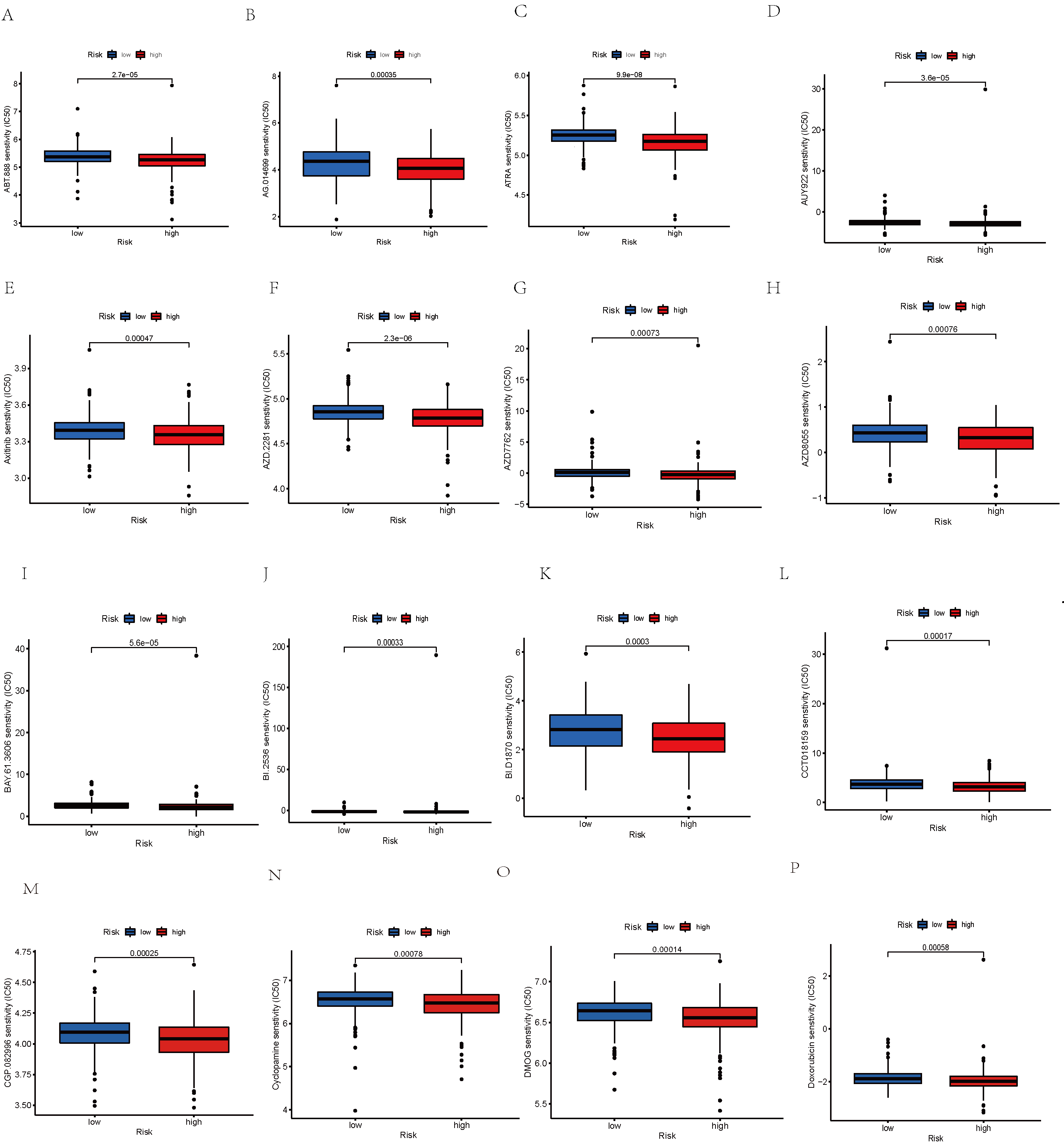

Supplement: Supplementary file 2 — Additional file 2. [file 12864_2023_9481_MOESM2_ESM.zip › Figure S6-1.tif]

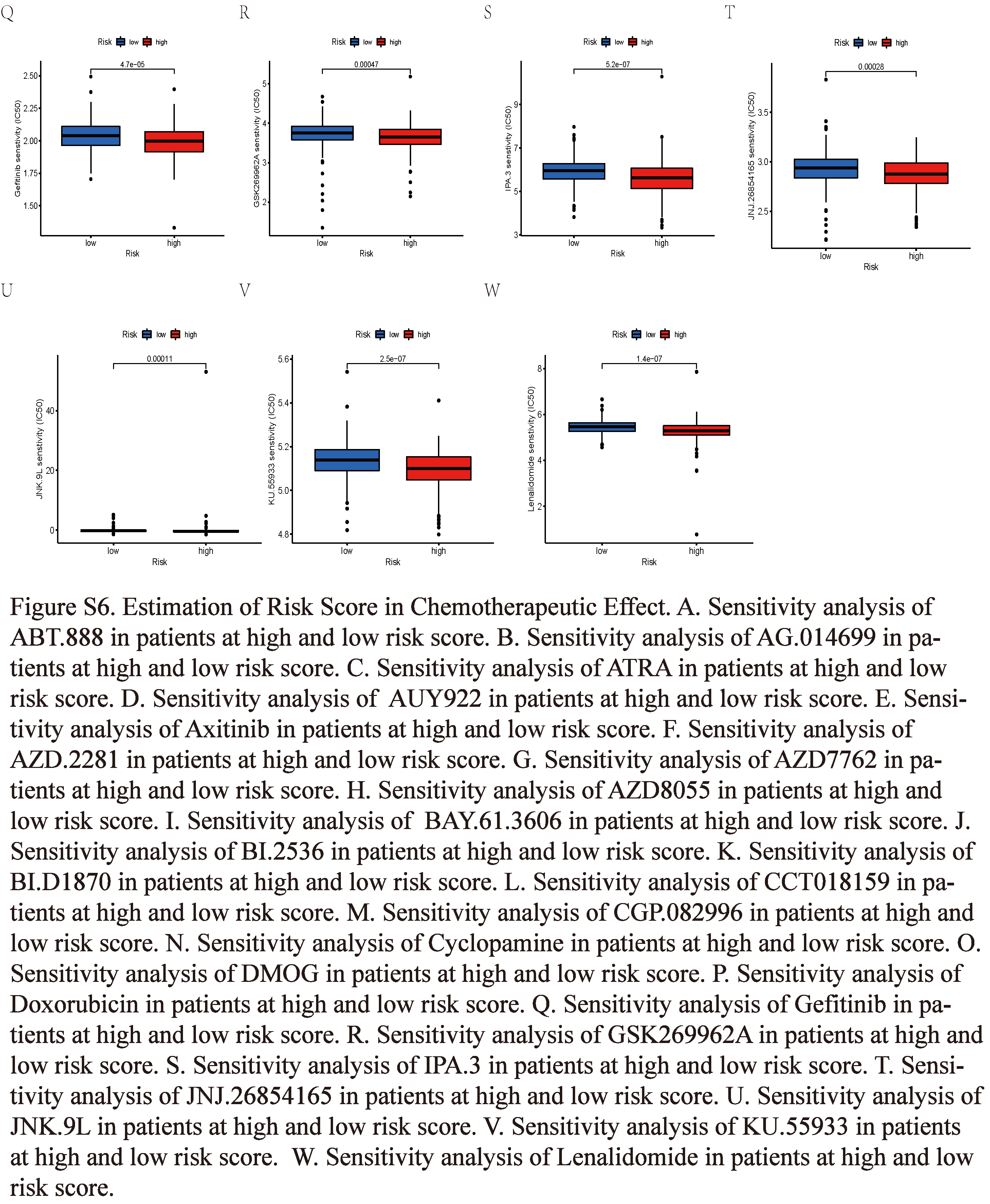

Supplement: Supplementary file 2 — Additional file 2. [file 12864_2023_9481_MOESM2_ESM.zip › Figure S6-2.tif]

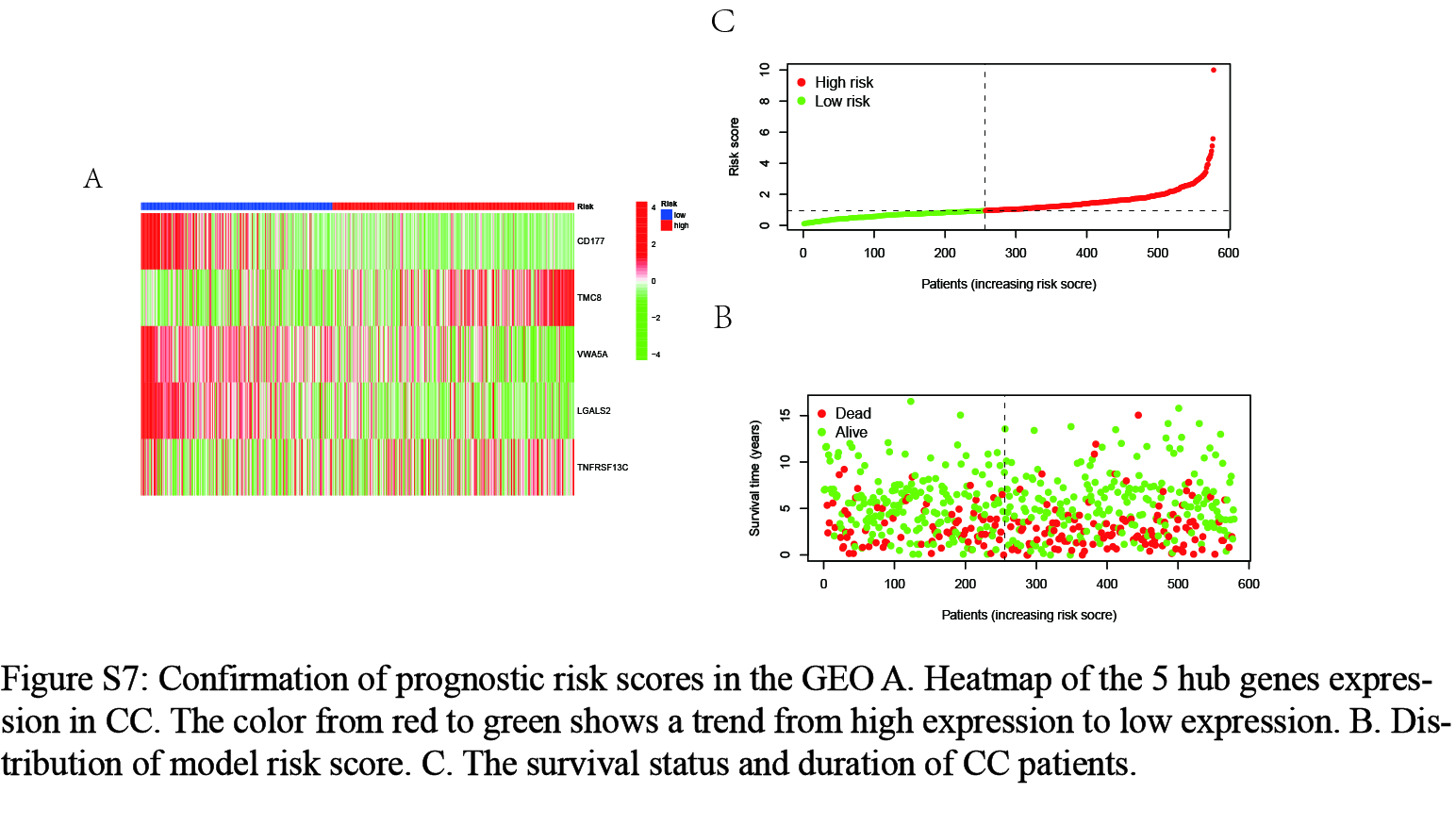

Supplement: Supplementary file 2 — Additional file 2. [file 12864_2023_9481_MOESM2_ESM.zip › Figure S7.tif]

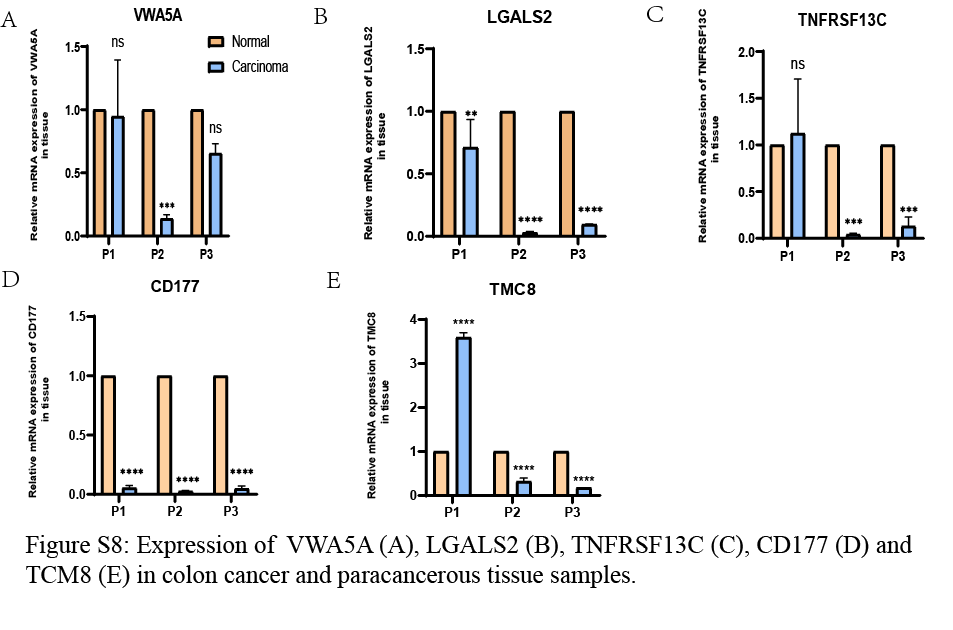

Supplement: Supplementary file 2 — Additional file 2. [file 12864_2023_9481_MOESM2_ESM.zip › Figure S8-1.tif]

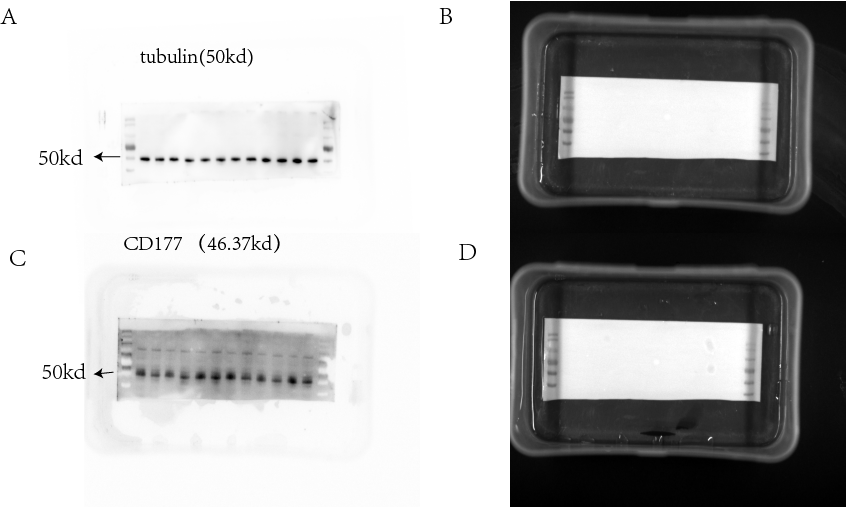

Supplement: Supplementary file 2 — Additional file 2. [file 12864_2023_9481_MOESM2_ESM.zip › Figure S9.tif]
